# A Prospective, Single Arm, Multi-Center Study Evaluating the Clinical Outcomes of Ventral Hernias Treated with OviTex^®^ 1S Permanent Reinforced Tissue Matrix: The BRAVO Study 12-Month Analysis

**DOI:** 10.3390/jcm10214998

**Published:** 2021-10-27

**Authors:** George DeNoto, Eugene P. Ceppa, Salvatore J. Pacella, Michael Sawyer, Geoffrey Slayden, Mark Takata, Gary Tuma, Jonathan Yunis

**Affiliations:** 1 Department of General Surgery, St. Francis Hospital, Roslyn, NY 11576, USA; 2 Department of Surgery, Indiana University School of Medicine, Indianapolis, IN 46202, USA; eceppa@iupui.edu; 3 Scripps Clinic and Scripps M.D. Anderson Cancer Center, Division of Plastic and Reconstructive Surgery, San Diego, CA 92130, USA; Pacella.Salvatore@scrippshealth.org; 4 Department of Surgery, Comanche County Memorial Hospital, Lawton, OK 73505, USA; michael.sawyer@ccmhhealth.com; 5 St. Luke’s Surgical Specialists, St. Luke’s Health System, Overland Park, KS 66213, USA; gslayden@saint-lukes.org; 6 Scripps Clinic Medical Group, Department of Surgery, La Jolla, CA 92037, USA; Takata.Mark@scrippshealth.org; 7 Capital Health Medical Group, Department of Plastic Surgery, Pennington, NJ 08534, USA; GTuma@capitalhealth.org; 8 Hernia Specialist, Center for Hernia Repair, Sarasota, FL 34239, USA; jpy@comcast.net

**Keywords:** ventral hernia repair, reinforced tissue matrix, decellularized extracellular matrix, recurrence

## Abstract

Background: Conflicting results from previous studies have led to dissent over whether surgical mesh is safe and effective in ventral hernia repair. A newer class of mesh known as a reinforced tissue matrix, combining a biologic scaffold and minimal polymer reinforcement, offers promise in reducing inflammatory response and increasing abdominal wall support. This study sought to assess the clinical utility of a reinforced tissue matrix (OviTex) in ventral hernia repair 12 months after implantation. Methods: This is a prospective, single-arm, multi-center study to evaluate the clinical performance of OviTex^®^ 1S Permanent (OviTex) in the repair of primary or recurrent ventral hernias (VH) in consecutive patients (ClinicalTrials.gov/NCT03074474). The rate of surgical site occurrences (SSOs) was evaluated 90 days post-surgery as the primary endpoint. Hernia recurrence and the incidence of postoperative events were evaluated between three and 12 months as secondary endpoints. The incidence of other complications and patient-reported outcomes were also recorded. Results: Ninety-two (92) patients were enrolled in the study, of whom seventy-six (76) reached the 12-month follow-up. All patients were at least 18 years of age with a BMI of <40 kg/m^2^. Hernia defects were <20 × 20 cm, classified as class I–III according to the CDC wound classification system. Of the 76 patients who reached 12-month follow-up, twenty-six (34%) had previous VH repairs and thirteen (17%) had previous surgical infection. Sixty (79%) had factors known to increase the risk of recurrence. Twenty patients (26%) experienced SSOs, with ten (13%) requiring procedural intervention. Two of the 75 patients (2.7%) experienced a recurrence. Conclusions: The low rate of hernia recurrence and SSOs requiring intervention illustrates the potential that reinforced tissue matrices, and OviTex 1S, in particular, have to improve outcomes in VH repairs. Follow-up to 24 months is ongoing.

## 1. Introduction

Ventral hernias occur when a defect in the abdominal wall allows the protrusion of organs and tissues outside of the abdomen [1]. Ventral hernias impair quality of life as they are painful and can prevent the patient from completing daily activities. In addition, ventral hernias can become life threatening if important abdominal organs, such as the intestine, become strangulated, causing blood supply to be cut off and the affected area of the organ to die [2]. While some hernias can be corrected by ‘reducing’ them or gradually pushing the protrusion back into the abdominal cavity, most ventral hernias require surgical intervention.

Surgical methods to correct ventral hernias have evolved over time. Simple suturing to close the defect proved unsatisfactory as it was associated with high recurrence rates. To ameliorate this problem, prosthetic supports began to be utilized in the 1960s. The use of synthetic surgical mesh to reinforce incisional hernia repairs was found to greatly reduce the risk of recurrence over the traditional suture repair [3,4]. Studies by Luijendijk et al. and Burger et al. showed that the use of mesh led to a reduction in hernia recurrence rates after 10 years from 63% for suture repair to 32% for synthetic mesh repair [3,4]. However, this success came at a cost of an increase in complications caused by the synthetic mesh, including bowel obstruction, fistula, and infection [3]. Kokotovic et al. have shown that the incidence of complications caused by synthetic meshes, severe enough to require surgical treatment, progressively increases over long-term follow-up [5]. In a Danish hernia registry, the incidence of reoperations to treat these mesh complications was shown to increase by approximately 1% per year for a period of at least seven years [5,6]. These results are comparable to those published by Hawn et al., who followed 1071 permanent prosthetic repairs for a median of 60.6 months and observed that synthetic meshes required explantation at a rate of 5.1% [7]. The high surgical site infection rates of synthetic meshes directly correlate with the need for explantation [7]. Synthetic mesh-related complications and the need for explantation are also attributed to the chronic inflammation associated with these materials, which has been demonstrated to persist in some cases for at least eight years following repair [8]. Due to these issues, alternative meshes, which would not cause high infection and inflammatory rates, were sought.

One advancement was the creation of synthetic meshes that resorbed over time. These resorbable synthetics aimed to provide reinforcement during the post-surgery healing phase and subsequently degrade to reduce the risk of persistent inflammation and the associated complications seen with permanent synthetics. They are made from polymers such as polyglycolic acid (PGA), trimethylene carbonate (TMC), polylactic acid (PLA), or combinations thereof, and more recently poly-4-hydroxybutyrate (P4HB). The degradation and resorption profiles of these materials differ, taking anywhere from six weeks to three years to fully absorb. However, as the degradation and resorption of the polymers progress, the polymer loses its strength. Depending on the material, resorbable polymers retain sufficient strength to solely support the repair for anywhere from 16–50% of their complete resorption time [9,10]. The ability of the surgical site to heal and host tissue to form over the mesh is dependent on the material composition of the mesh and the host immune response and inflammatory reaction [11]. The integrity of the tissue formed over the surgical site therefore varies and historically these mesh types have shown high recurrence rates [12,13,14]. However, these meshes are relatively new and have shown some promising results in initial publications [12,13,14]. Long-term results are not yet available, and the efficacy and safety of resorbable meshes continue to be evaluated in several clinical studies [12,15,16].

Another mesh alternative is biological meshes that are made from human- or animal-derived connective tissue known as the ‘extracellular matrix’. These meshes are made from dermis, small intestine, bladder, pericardium, and other organs rich in connective tissue. The perceived advantages are that biologic meshes remodel into a natural collagen layer, do not cause an extended inflammatory response, may be left in situ in the presence of an infection, and are believed to be better suited for use in contaminated fields. Because of long-term laxity and especially cost, use has been limited to more complex patients [13,14]. In addition, there remains a debate on whether biologics deliver their stated benefits. In a study comparing synthetic and biologic meshes in single-stage repair in patients with contaminated surgical fields, synthetic meshes were found not to be inferior to biologic meshes, and the price difference between the synthetic and more costly biologics could not be justified [17]. In contrast, a study by Garvey et al., which analyzed the use of biologics in 191 complex abdominal wall reconstruction patients with a minimum follow-up of 36 months, showed certain biologic meshes to be superior to traditional synthetics [18]. Excluding patients who underwent bridged repair, recurrence rates were similar to those seen with synthetic meshes, but the infection and explantation rates were lower [19]. The only biologic not found to provide these benefits was the human acellular dermal matrix product [18]. These findings suggest that the reported incidence of complications associated with the use of biologic meshes may be in part due to their predominant use in more challenging situations. Biologic meshes are therefore a promising alternative to traditional synthetic meshes.

**Figure 1:**
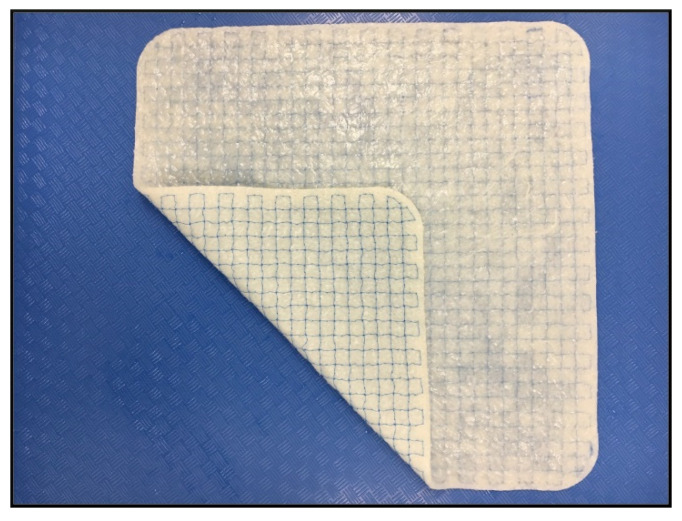
Photograph of OviTex 1S Permanent RTM.

Hybrid meshes, combining both synthetic and biologic materials, are another recent surgical option. One such mesh is OviTex 1S Permanent, a reinforced tissue matrix (RTM). This product aims to preserve the benefits of both its synthetic and biologic components while minimizing their downsides. RTMs consist of layers of extracellular matrix embroidered with polymer reinforcement. The biologic component of OviTex is derived from ovine rumen; sheets of this biologic are combined in several thicknesses (strengths) and reinforced using a monofilament polypropylene thread. In OviTex 1S Permanent, six layers are sewn together and sterilized (Figure 1). Prior to clinical introduction, the biological performance of OviTex was studied and compared to a variety of synthetic and biologic meshes in a non-human primate abdominal wall defect model [11]. It was found that OviTex 1S invoked less of an inflammatory response and promoted better wound healing than the synthetic meshes, which caused the formation of scar-like tissue [8]. OviTex 1S also better maintained its structural integrity and repair geometry, a primary issue with many biologics [8]. Based on the results of the primate study, it was hypothesized that the use of OviTex 1S in ventral hernia repair (in comparable subjects) would result in similar or fewer hernia recurrences and incidence of surgical site occurrences (SSOs), such as seroma formation and infections, than traditional synthetics. In addition, it was postulated that the incidence of related secondary procedures, including fluid aspirations, wound debridement, and explantations, would also be lower in patients treated with OviTex 1S compared to traditional synthetics [18,20,21,22].

To determine if the use of OviTex 1S in ventral hernia repair resulted in lower rates of recurrence, SSOs, secondary procedures, and explantation compared to historical rates for traditional synthetics, we conducted a prospective, multi-site, single-arm clinical study over a period of 12 months. 

## 2. Materials and Methods

### 2.1. Study Design

This prospective, single-arm, multi-center study evaluates the clinical performance of OviTex 1S Permanent (designed by TELA Bio, Malvern, PA, USA) for primary or recurrent ventral hernia repair (ClinicalTrials.gov/NCT03074474). This study was designed to treat up to one hundred (100) patients at seven (7) investigational sites throughout the United States. The protocol was approved by each center’s Institutional Review Board (IRB) prior to enrolling subjects. Informed consent was obtained from all individual participants included in the study. Endpoints were evaluated at days 30 and 90 and 12 months.

### 2.2. Inclusion/Exclusion Criteria

Included were subjects 18 years of age or older and suffering from uncomplicated ventral hernias that required surgical repair (open, laparoscopic, or robotic) by use of mesh of expected size 18 cm × 22 cm, 20 cm × 20 cm, or less. Subjects meeting Centers for Disease Control and Prevention (CDC) SSI Wound Classification Class I (Clean), Class II (Clean–Contaminated), or Class III (Contaminated) were included. Subjects meeting the ventral hernia working group (VHWG) grade I (low risk), grade II (co-morbid), or grade III (potentially contaminated) were also included. Excluded were patients with a BMI above 40 kg/m^2^, CDC/SSI Wound Classification Class IV (Dirty-Infected), VHWG grade IV, and those who required an implant > 20 cm × 20 cm, were pregnant, had a life expectancy of <2 years, had a recent history of drug or alcohol abuse in the last 3 years, or had an allergy to ovine-derived products. 

### 2.3. Surgery

Ventral hernia repair using OviTex 1S Permanent was performed at seven institutions. Surgical preparation regimens were performed per surgeons’ individual institutional guidelines. Surgeons were free to choose both the approach as well as the plane of placement for completing the procedures. At the time of surgery, surgical technique, defect size, implant size, and anatomical placement were recorded. Operative data including component separation, fixation method, number and placement of drains, rectus muscle condition, presence or absence of adhesions, concomitant procedures, iatrogenic injury, blood loss, skin closure, and operative time were also recorded. Surgeons used their discretion to determine adequate mesh size for defect coverage and were able to cut the mesh to a smaller size. Using a Likert scale, surgeons were asked to provide their subjective assessment on mesh handling characteristics as defined by ease of placement and ease of securing the implant. 

### 2.4. Follow-Up

Post-operative patient follow-up was conducted at 30 and 90 days and 12 months post-surgery. At each visit, the patient underwent physical examination to assess any post-operative adverse events, surgical complications, or hernia recurrence at the site of hernia repair. Any clinical suspicion of recurrent hernia was evaluated by abdominal CT scan. In addition, patient-reported outcome data were evaluated using the HerQLes Quality of Life (QoL) survey.

### 2.5. Primary Endpoint 

The primary endpoint is the incidence of procedure- and implant-related post-operative adverse events occurring within the first three months of ventral hernia repair. This consisted of surgical site occurrences such as ileus, deep or superficial wound infection, seroma, hematoma, wound dehiscence, skin necrosis, and fistulae. Other complications recorded included ileus, bowel obstruction, and deep venous thrombosis.

### 2.6. Secondary Endpoints

The secondary endpoints are hernia recurrence at the site of the repair, confirmed by CT scan, and the incidence of post-operative adverse events and surgical complications noted at the hernia repair site occurring at time points later than three months after the index surgery. These consisted of surgical site occurrences such as ileus, deep or superficial wound infection, seroma, hematoma, wound dehiscence, skin necrosis, and fistulae. Other procedure complications recorded included ileus, bowel obstruction, and deep venous thrombosis. The HerQLes QoL survey was used for a disease-specific assessment. 

### 2.7. Statistical Analysis

All results are mean (SEM). To estimate the chance of patients surviving without a hernia recurrence, a Kaplan–Meier survival analysis was performed. To determine the change in patient HerQLes scores before and after ventral hernia repair surgery, a 1-way ANOVA was performed followed by Dunn’s multiple comparison post hoc test to calculate difference in score between baseline and post-op time points. To determine if there was a difference in EQ-5D or HerQLes scores between 90 days and 12 months, a Wilcoxon matched-pairs signed rank test was performed. *p* < 0.05 was considered statistically significant. All analyses were performed in GraphPad Prism 9.

## 3. Results

### 3.1. Ninety-Day Outcomes

Ninety days after ventral hernia repair, 84 of the 92 patients (91.3%) enrolled in the study were evaluated. The loss of eight patients occurred as four patients died from unrelated causes, one withdrew consent, and three were excluded as screening failures prior to implantation. The population at 90 days post-op had an average BMI of 30.8 kg/m^2^, 46 (55%) were obese, 31 (37%) had a prior ventral hernia repair with an average of two repairs per patient, 14 (17%) had a history of surgical infection, and 63 (75%) had a VHWG grade II or above (Table 1). Open ventral hernia repair was the most common approach, performed in 55 patients (65%), and laparoscopic and robotic repairs were performed in 11 (13%) and 18 (21%) patients, respectively (Table 2). Primary closure was achieved in 79 patients (93%), with 43 (51%) requiring component separation (Table 2). Twenty-one (21/84, 25%) patients experienced an SSO, of which twelve (12/84, 15%) were a surgical site infection. Of those SSOs, eleven patients (11/84, 13%) required a procedural intervention (PI) in the form of a percutaneous drainage (Table 3). There was one surgical intervention in a patient where part of the mesh was removed four weeks post-surgery due to an infection related to a colon perforation. This patient continued to be followed throughout the study. There were no recurrences in the first 90 days. 

### 3.2. Twelve-Month Outcomes

**Figure 2:**
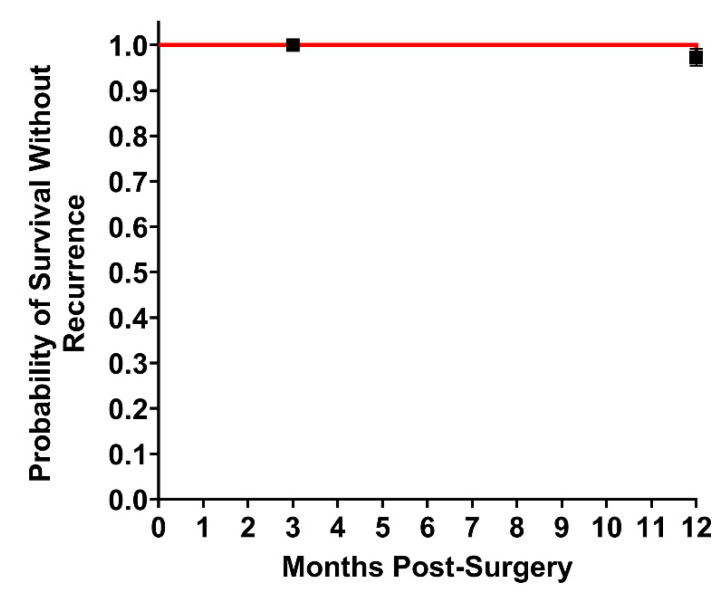
Probability of Hernia-Free Survival. Kaplan–Meier estimate of probability of hernia recurrence in the patient population indicated by Kaplan-Meier curve (red line). Fraction of patients with hernia recurrence 3 and 12 months post-surgery. *n* = 75. Standard error.

Twelve months after surgery, 76 of the original 92 patients enrolled were able to be evaluated. In addition to the aforementioned eight patients lost before the 90-day follow-up, an additional five patients were lost for 12-month follow-up and three patients withdrew consent. The population at 12 months post-op had an average BMI of 31 kg/m^2^, 44 (58%) were obese, 26 (34%) had a prior ventral hernia repair with an average of two repairs per patient, 13 (17%) had a history of surgical infection, and 60 (79%) had a VHWG grade II or above (Table 1). Sixty (79%) had one or more factors reported to increase the risk of recurrence [15] and two patients had unrelated malignancies (Table 4). Open ventral hernia repair was the most common approach, performed in 47 patients (62%), and laparoscopic and robotic repairs were performed in 11 (14%) and 18 (24%), respectively (Table 2). Primary closure was achieved in 70 patients (92%), with 41 (54%) requiring component separation (Table 2). Twenty patients (20/76 26%) experienced an SSO over the course of 0 days to 12 months. Seventeen of these occurred between 0 and 90 days and three occurred between 90 days and 12 months (Table 3). Thirteen of the patients (13/76, 17%) had an SSO classified as a surgical site infection. Ten of the patients (10/76, 13%) required a procedural intervention (PI) in the form of wound debridement or percutaneous drainage. One patient developed an enterocutaneous fistula five months post-operation, underwent takedown and had their mesh removed. Two patients experienced recurrences (2 of 75; 2.7%) (Figure 2). A Kaplan–Meier analysis estimated a 100% chance of not having a recurrence at 3 months post-surgery and a 97.3% chance of not having a recurrence at 12 months post-surgery (Figure 2). One of the recurrences occurred in a patient with an umbilical hernia and a diastasis at nine months post-surgery. The diastasis was plicated during the original surgery, and the recurrence was located adjacent to the original repair in the diastasis. Inspection of the original construct during the surgery to repair the recurrence and the diastasis showed the original repair to be intact, vascularized, and fully integrated. The second recurrence presented as a parastomal hernia occurring at the superior aspect to the original study repair.

### 3.3. Quality of Life Survey

**Figure 3:**
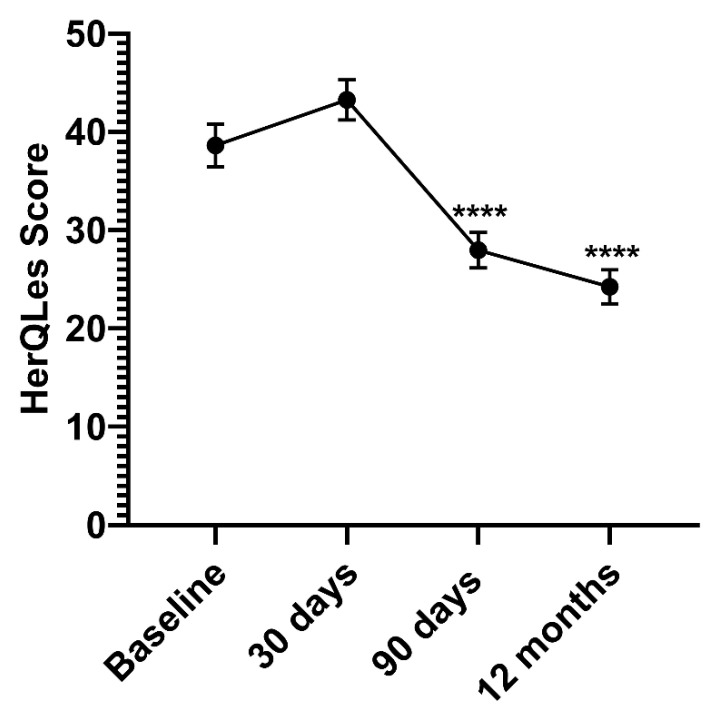
HerQLes Summary Scores Before and After Ventral Hernia Repair. Patient HerQLes summary scores before surgery (Baseline), 30 days post-op, 90 days post-op, and 12 months post-op. Lower HerQLes summary score indicates better QOL. *n* = 63. Values are mean ± SE. **** *p* < 0.0001.

All patient-reported outcome scales showed improvements from the preoperative baseline at both timepoints, with the most improvement seen at 12 months. The hernia-specific patient-reported outcome quality of life survey, the HerQLes, showed the most pronounced improvement; the total score at 12 months was lowered by 39%, indicating an improvement in quality of life after hernia repair (Table 5) (Figure 3). HerQLes summary scores, representing all 12 questions, were calculated so that 100 was the worst possible score and 0 was the best possible score. The summary scores were found to be significantly lower at 90 days and at 12 months in comparison to starting summary scores before surgery, at baseline, indicating improved quality of life (Figure 3). Even at 30 days, the time period during which most complications and recurrences occur, the summary score was not significantly increased in comparison to baseline, showing that there was no decrease in quality of life at any timepoint (Figure 3). 

## 4. Discussion

This prospective, multi-center study presents the 12-month results of the most widely used RTM, OviTex 1S Permanent. The study aimed to reflect, as much as possible, routine clinical practice, with no restrictions regarding the choice of surgical technique or plane of placement of the RTM implant. The main exclusions were patients with a BMI of greater than 40 kg/m^2^ and those with CDC Wound Class IV. 

The results presented here at one year show a low incidence of hernia recurrence following a repair with OviTex 1S Permanent RTM. Observations made regarding the two recurrences, which were both diagnosed around nine months after the index surgery, demonstrated that these were not due to a failure of the RTM construct. Both occurred directly adjacent to the original repair, while the original repairs remained intact. One underwent a surgical repair during which the RTM was found to be well vascularized and integrated and the other did not require surgical treatment. The incidence of recurrence of 2.6% at 12 months is lower than that reported in publications describing prospective studies for other materials, whether permanent synthetic, resorbable synthetic, or biologic [12,15,21,23,24]. The range of recurrence rates in these studies spanned from 9% to 28% [12,15,21,23,24]. 

Repair with OviTex 1S Permanent RTM also resulted in a low incidence of SSOs and surgical site infections (SSIs). The majority of procedure-related adverse events occur within the first 90 days post-operation. During this critical time period, we observed an SSO incidence rate of 25%. Only three additional patients experienced an SSO between 90 days and 12 months. In comparison to studies using other meshes, the overall rate appears to be in line if not lower than the rates reported for these other meshes, which range from 28% to 31% [12,21,24]. Of the 76 patients followed up at one year, 13 (17%) experienced an SSI and none developed infection of the RTM. We believe that the monofilament nature of the prolene reinforcement surrounded by neovascularized biologic tissue offers the mesh some protection from infection. SSOs and SSIs are considered to be associated with an increased risk of recurrence. This makes the low rate of recurrence observed with OviTex 1S Permanent RTM in a predominantly higher risk population (VHWG 2–3) requiring component separation in greater than 50% of the patients all the more remarkable given that the incidence of SSOs and SSIs was comparable to some of the other types of meshes [12,15]. This counterintuitive observation supports the one made by Ceppa et al., who, in a recent study, found that the presence of SSOs in patients treated with a coated synthetic mesh was associated with hernia recurrence in more than 50% of the patients, whereas the presence of an SSO in OviTex RTM-treated patients was associated with a hernia recurrence in less than 20% of the patients, a difference that was statistically significant [25]. 

Additional surgical intervention rates were also very low for repair with OviTex. By 12 months, only one of the SSOs recorded required a reoperation. In this case, the surgical treatment of a fistula required the removal of the RTM. A reoperation rate of one patient out of 76 is only 1.3%; this is lower than reoperation rates found in the literature for other mesh types, which range from 11.6–19% [21,24]. 

As the surgeons were able to choose the surgical technique and approach, these positive outcomes for use of OviTex are therefore relevant independent of surgery type. Compared with several other clinical studies such as the COBRA and Roth studies [12,24], the surgical techniques and planes of placement in this study are more heterogeneous and include approximately 40% of patients in whom the implant was placed as an intraperitoneal underlay. Some publications have found that the retromuscular sublay position, primarily used in other clinical studies, exhibits fewer complications and recurrences than other locations [26]. However, a recent analysis based on the Abdominal Core Health Quality Collaborative (ACHQC) registry found that, while confirming the trend towards more retromuscular placements, there was no significant difference in the incidence of complications and recurrences between these locations [23]. The data described here so far support that conclusion. Thus, OviTex was associated with low rates of complications and recurrences regardless of surgical technique or approach. 

These 12-month results support the design philosophy of OviTex RTMs as a new class of reinforced materials for hernia repair that incorporate the positive features of synthetic and biologic materials, while minimizing their negative characteristics. Important to the success of a biologic material is the preservation of the ECM’s matrisome after processing. OviTex RTMs have been characterized to retain 153 unique macromolecules, which promote cell signaling as well as the assembling and remodeling of the ECM [27]. The biologic component provides a natural scaffold for healing that is remodeled into a new layer of functional collagen, morphologically distinctive from scar tissue [11]. Reducing the amount of synthetic material lowers the associated dose-dependent inflammation that can lead to both short- and long-term complications [5,28]. Together, these two systems contribute to the biomechanical properties of OviTex RTMs, which have been purposely designed to match those of the human abdominal wall, and are believed to contribute to the observed low rate of recurrence [29]. The result is an alternative solution for ventral hernia repair that preserves the polymer reinforcement provided by synthetic materials, while minimizing the amount of synthetic material used.

In addition to positive patient outcomes, surgeons also had a positive opinion on the use of OviTex 1S Permanent RTM. The investigators were asked to evaluate the ease of use of the material on a 5-point Likert scale ranging from ‘very easy’ to ‘very difficult’. For every procedure, all users considered OviTex 1S Permanent RTM to be either ‘very easy’ or ‘easy’ to shape, place, and fixate, irrespective of the plane of placement chosen, whether they used sutures or tacks, or whether the procedure was performed open or minimally invasively. It appears the use of OviTex 1S Permanent RTM is therefore beneficial for both surgeons and patients.

Continued evaluation of OviTex 1S Permanent RTM is important to definitively conclude its optimal uses as well as its long-term safety and efficacy. The patients included in this study are continuing to be evaluated up to 24 months post-surgery. Thus far, the safety and efficacy of OviTex shown in this original 12-month study have been corroborated as there have been no additional recurrences in the 51 patients for whom 24-month follow-up has been completed. Future studies will focus on other indications of OviTex such as effectiveness in robotic ventral hernia repair. In this way, the potential benefits of the OviTex hybrid mesh structure will be determined. 

This study is limited by sample size and longevity. The results presented here are only representative of 12 months post-follow-up, which is relatively short. However, follow-up to 24 months is ongoing. The sample size of patients is relatively low; however, it is still robust enough to perform statistical analyses. The authors are striving to maintain all 76 patients followed to 12 months to 24 months as well in order to have the same statistical power. Additionally, the assessment of the status of the repair was based on history and clinical exam, and imaging modalities were only used to assess the status of the repair in cases where re-herniation was suspected. Finally, this study is a prospective single-arm without a direct randomized control. Given the heterogeneity of ventral hernia patients and allowing that each study has its own unique design, comparisons with results published in the literature should be made with caution. In addition, only a select set of endpoints were measured, leaving certain comparisons to published literature not possible. 

## 5. Conclusions

In conclusion, the 12-month results of this prospective multi-center study reveal good clinical results and patient outcomes obtained with OviTex 1S Permanent RTM, comparable to and in some cases better than those reported for other mesh materials. Specifically, the recurrence rate appears to be in line or lower than reported in studies with similar populations in the literature [30,31,32,33]. In our experience, the addition of the polymer to the biologic mesh does not appear to increase the risk of mesh infection. In addition, the occurrence of only two recurrences in 75 patients at 12 months represents an encouraging trend. Twenty-four-month follow-up of the patients is continuing. 

## Figures and Tables

**Table 1:** Preoperative Variables, Comorbid Conditions, and Perioperative Variables.

Preoperative Variables	90 Days	12 Months
Subjects Enrolled—92		
Subjects Included, *n* (%)	84 (91%)	76 (83%)
Sex, *n* (%)		
Male	34 (40%)	31 (41%)
Female	50 (60%)	45 (59%)
Age (years), range	62 (33.8–84.8)	62 (33.8–84.8)
Body Mass Index (kg/m^2^), mean (range)	31 (19.5–39.7)	31 (21.6–39.5)
Comorbid Conditions		
Obesity, *n* (%)	46 (55%)	44 (58%)
Patients with Prior VH Repairs, *n* (%)	31 (37%)	26 (34%)
Number of repairs, mean	2 (0%)	2 (0%)
Prior SSI, *n* (%)	4 (5%)	3 (4%)
History of Surgical Infection, *n* (%)	14 (17%)	13 (17%)
Perioperative Variables		
CDC Wound Class, *n* (%)		
CDC Class I Clean	68 (81%)	61 (80%)
CDC Class II Clean–Contaminated	12 (14%)	11 (14%)
CDC Class III Contaminated	4 (5%)	4 (5%)
VHWG, *n* (%)		
Grade I	21 (25%)	16 (21%)
Grade II	44 (52%)	43 (57%)
Grade III	19 (23%)	17 (22%)

CDC: Centers for Disease Control and Prevention; VHWG: Ventral Hernia Working Group.

**Table 2:** Operative Characteristics.

Operative Characteristics	90 Days	12 Months
Hernia Defect Size (cm^2^), mean (range)	101 (1–384)	101 (1–384)
Mesh Size at Implantation (cm^2^), mean (range)	279 (30–400)	277 (30–400)
Approach, *n* (%)		
Open	55 (65%)	47 (62%)
Laparoscopic	11 (13%)	11 (14%)
Robotic	18 (21%)	18 (24%)
Plane of Placement, *n* (%)		
Retrorectus	35 (42%)	29 (38%)
Intraperitoneal	38 (45%)	36 (47%)
TAR	9 (11%)	9 (12%)
Retrofascial/Pre-Peritoneal	1 (1%)	1 (1%)
Onlay	1 (1%)	1 (1%)
Primary Closure, *n* (%)	78 (93%)	70 (92%)
Component Separation, *n* (%)	43 (51%)	41 (54%)
Time in Surgery (Hours), mean (range)	2.62 (0.70–9.68)	2.62 (0.70–9.68)
Hospital Stay (Days), mean (range)	4.46 (0–18)	4.45 (0–18)

TAR: Transversus Abdominis Muscle Release.

**Table 3:** Primary and Secondary Endpoints: Adverse Events.

Adverse Events	90 Days	12 Months
Total Patients	84	76
Hernia Recurrence, *n (*%*)*	-	2 (3%)
SSO (patients), *n* (%)	21 (25%)	20 (26%)
Seroma (requiring intervention)	3 (4%)	1 (1%)
Hematoma	4 (5%)	4 (5%)
Wound Dehiscence	1 (1%)	1 (1%)
Skin Necrosis	1 (1%)	1 (1%)
Fistulae	2 (2%)	2 (3%)
Superficial Infection	8 (10%)	7 (9%)
Deep/Abscess Infection	4 (5%)	6 (8%)
Organ Space Infection	-	-
SSO/SSI Requiring Procedural Intervention	11 (13%)	10 (13%)
Complications		
Bowel Obstruction	1 (1%)	1 (1%)
DVT/PE	3 (4%)	3 (4%)
Ileus	7 (8%)	6 (8%)
Malignancy	1 (1%)	2 (3%)
Any other non-surgery or hernia-related complications	15 (18%)	15 (20%)
Full Mesh Removal	*	1 (1%)

* Partial mesh removal at 90 days. SSO: Surgical Site Occurrence; SSI: Surgical Site Infection; DVT: Deep Vein Thrombosis; PE: Pulmonary Embolism.

**Table 4:** Factors for High Risk of Complications, One-Year Cohort.

Risk Factor	Number of Patients
BMI between 30 and 40	44 (58%)
Active smokers	5 (7%)
COPD	3 (4%)
Diabetes mellitus	18 (24%)
Coronary artery disease	5 (7%)
Advanced age (>/=75 yrs)	9 (12%)
Total High Risk (at least one)	60 (79%)

BMI: Body mass index.

**Table 5:** Secondary Endpoints: Quality of Life Surveys.

Quality of Life Surveys	90 Days	12 Months
EQ-5D		
Change (Baseline to Time point), mean	−0.26	−0.91
Interpretation	2.3% improvement	11% improvement
Patients, *n*	76	69
HerQLes		
Change (Baseline to Time point), mean	−10.63	−15.46
Interpretation	25.9% improvement	38.8% improvement
Patients, *n*	75	69

## Data Availability

The datasets generated during and/or analyzed during the current study are available from the corresponding author on reasonable request.
